# TREM2 in neurodegeneration: evidence for association of the p.R47H variant with frontotemporal dementia and Parkinson’s disease

**DOI:** 10.1186/1750-1326-8-19

**Published:** 2013-06-21

**Authors:** Sruti Rayaprolu, Bianca Mullen, Matt Baker, Timothy Lynch, Elizabeth Finger, William W Seeley, Kimmo J Hatanpaa, Catherine Lomen-Hoerth, Andrew Kertesz, Eileen H Bigio, Carol Lippa, Keith A Josephs, David S Knopman, Charles L White, Richard Caselli, Ian R Mackenzie, Bruce L Miller, Magdalena Boczarska-Jedynak, Grzegorz Opala, Anna Krygowska-Wajs, Maria Barcikowska, Steven G Younkin, Ronald C Petersen, Nilüfer Ertekin-Taner, Ryan J Uitti, James F Meschia, Kevin B Boylan, Bradley F Boeve, Neill R Graff-Radford, Zbigniew K Wszolek, Dennis W Dickson, Rosa Rademakers, Owen A Ross

**Affiliations:** 1 Department of Neuroscience, Mayo Clinic, Jacksonville, FL, USA; 2 Dublin Neurological Institute at the Mater Misericordiae University Hospital, Conway Institute of Biomolecular & Biomedical Research, University College Dublin, Dublin, Ireland; 3 Department of Clinical Neurological Sciences, Schulich School of Medicine and Dentistry, The University of Western Ontario, London, Ontario, Canada; 4 Department of Neurology, University of California, San Francisco, CA, USA; 5 Department of Pathology and Alzheimer's Disease Center, University of Texas Southwestern Medical Center, Dallas, TX, USA; 6 Cognitive Neurology & Alzheimer Disease Center, Northwestern University Feinberg School of Medicine, Chicago, IL, USA; 7 Department of Neurology, Drexel University College of Medicine, Philadelphia, PA, USA; 8 Department of Neurology, Mayo Clinic, Rochester, MN, USA; 9 Department of Neurology, Mayo Clinic, Scottsdale, AZ, USA; 10 Department of Pathology and Laboratory Medicine, University of British Columbia, Vancouver, Canada; 11 Department of Neurology, Medical University of Silesia, Katowice, Poland; 12 Department of Neurology, Jagiellonian University, Krakow, Poland; 13 Department of Neurodegenerative Disorders, Medical Research Centre, Polish Academy of Sciences, Warsaw, Poland; 14 Department of Neurology, Mayo Clinic, Jacksonville, FL, USA

**Keywords:** TREM2, Frontotemporal dementia, Parkinson disease, Genetic association

## Abstract

**Background:**

A rare variant in the Triggering Receptor Expressed on Myeloid cells 2 (*TREM2*) gene has been reported to be a genetic risk factor for Alzheimer’s disease by two independent groups (Odds ratio between 2.9-4.5). Given the key role of TREM2 in the effective phagocytosis of apoptotic neuronal cells by microglia, we hypothesized that dysfunction of TREM2 may play a more generalized role in neurodegeneration. With this in mind we set out to assess the genetic association of the Alzheimer’s disease-related risk variant in *TREM2* (rs75932628, p.R47H) with other related neurodegenerative disorders.

**Results:**

The study included 609 patients with frontotemporal dementia, 765 with amyotrophic lateral sclerosis, 1493 with Parkinson’s disease, 772 with progressive supranuclear palsy, 448 with ischemic stroke and 1957 controls subjects free of neurodegenerative disease. A significant association was observed for the TREM2 p.R47H substitution in susceptibility to frontotemporal dementia (OR = 5.06; p-value = 0.001) and Parkinson’s disease (OR = 2.67; p-value = 0.026), while no evidence of association with risk of amyotrophic lateral sclerosis, progressive supranuclear palsy or ischemic stroke was observed.

**Conclusions:**

Our results suggest that the TREM2 p.R47H substitution is a risk factor for frontotemporal dementia and Parkinson’s disease in addition to Alzheimer’s disease. These findings suggest a more general role for TREM2 dysfunction in neurodegeneration, which could be related to its role in the immune response.

## Introduction

The Triggering Receptor Expressed on Myeloid cells 2 (TREM2) protein is a member of the innate immune receptor of the TREM family. It is expressed on activated macrophages, immature dendritic cells, osteoclasts and microglia [1]. Recent findings support a model in which TREM2 suppresses inflammation while at the same time promoting tissue repair through the removal of apoptotic cells [2]. Homozygous loss of function mutations in *TREM2* cause a rare autosomal recessive disease known as polycystic lipomembranous osteodysplasia with sclerosing leukoencephalopathy (PLOSL), or Nasu-Hakola disease [3]. While the mechanisms of neurodegeneration in this disorder are not completely known, it was proposed that the lack of TREM2 impairs the clearance of apoptotic neurons by microglia, leading to the accumulation of necrotic debris [4].

The unique combination of presenile frontal-type dementia, caused by the demyelination of the central nervous system, with polycystic osseous lesions makes it easy to distinguish PLOSL from more typical forms of sporadic and familial frontotemporal dementia (FTD). However, Guerreiro et al. recently performed next-generation DNA sequencing in patients with behavioral variant FTD (bvFTD) without bone cysts and identified homozygous mutations in *TREM2*[5]. Another mutation affecting the 5’ consensus donor splice site in intron 1of *TREM2* had been previously implicated in early-onset dementia without bone cysts in a Lebanese family [6]. Moreover, a third study has now identified a homozygous TREM2 p.Y198X mutation in a family with autosomal recessive FTD from Columbia that co–segregates with disease, suggesting that *TREM2* homozygous mutations can cause a more typical FTD phenotype [7].

The interest in *TREM2* as a genetic risk factor for neurodegenerative diseases greatly increased when two recent studies independently identified a rare missense mutation (rs75932628, p.R47H) in the *TREM2* gene as an important risk factor for late-onset Alzheimer’s disease (AD). Jonsson et al. used whole-genome sequencing data obtained from 2261 Icelandic individuals to identify variants likely to affect protein function. Upon imputation of these variants into a series of late-onset AD patients and controls using genome-wide association data, they showed that the p.R47H variant conferred a significant, three-fold risk of late-onset AD. The association was replicated across additional AD-control series from Europe and the US. As part of an independent study by Guerreiro et al., we utilized exome and whole-genome sequencing in AD patients and identified an increased burden of *TREM2* variants in patients compared to controls [5]. The variants clustered in exon 2 of *TREM2* with the substitution p.R47H displaying the strongest association with risk of late-onset AD (odds ratio; OR ~4.5). Together these studies unequivocally implicated TREM2 p.R47H in AD risk; however its role in other neurodegenerative diseases has not yet been studied.

Given the possible function of TREM2 in the neuroimmune response and the phenotypic heterogeneity observed in carriers of *TREM2* mutations, we hypothesized that the TREM2 p.R47H substitution may be a general risk factor for neurodegenerative disorders. In the present study, we genotyped this variant in a series of well-characterized neurodegenerative patients with amyotrophic lateral sclerosis (ALS), FTD, Parkinson’s disease (PD), progressive supranuclear palsy (PSP), and ischemic stroke as well as a series of healthy controls.

## Results

Genotype and allele distributions were in Hardy–Weinberg equilibrium (HWE) in all cohorts. Table 1 displays the carrier frequency, odds ratios (OR), 95% confidence intervals (CIs), and p-values for each disease cohort in comparison to the controls series. We observed a carrier frequency of 0.45% (6/1324) in our North American control series which is similar to the frequency reported in our independent controls used for our previous study of TREM2 p.R47H in AD (0.37%; 15/4061) [5]. Using our control series (n=1324), we identified a significant association with the rs75932628 (p.R47H) variant in both the North American FTD series (OR = 5.06; p-value = 0.0012) and the North American PD series (OR = 3.14; p-value = 0.033). In the FTD series the association was observed to be stronger in the clinical series (OR = 5.46; p-value=0.0009) compared to the pathologic-confirmed cases with frontotemporal lobar degeneration with TDP-43 pathology (FTLD-TDP, OR = 3.56, p=0.14); however this could be, at least in part, related to the smaller autopsy series (n=132). A second FTD series was unavailable for replication.

**Table 1:** **Association analysis of *TREM2 *variant c.140G>A (rs75932628; p.R47H) in disease**

**Disorder (*n *)**		**Patient, *n *(%)**		**Control, *n *(%)**		**OR (95% CI)**		**p-value**^**a**^
Controls	(1324)			6	(0.45)			
**FTD**	**(609)**	**13**	**(2.1)**	**6**	**(0.45)**	**5.064**	**(1.9, 13.51)**	**0.0012**
ALS	(765)	5	(0.7)	6	(0.45)	1.466	(0.43, 4.94)	0.5378
**PD**	**(683)**	**9**	**(1.3)**	**6**	**(0.45)**	**3.144**	**(1.1, 9.03)**	**0.0333**
PSP	(722)	5	(0.6)	6	(0.45)	1.537	(0.41, 5.78)	0.5249
Ischemic Stroke	(448)	3	(0.7)	6	(0.45)	1.506	(0.37, 6.17)	0.5689

^a^ Uncorrected p-values are shown. The association in FTD would remain significant after a conservative Bonferroni correction for multiple testing (significance level p<0.01).

In an attempt to replicate the association of p.R47H with PD we studied two additional patient-control series of European Caucasian descent from Ireland and Poland. Although the association analysis was not statistically significant in these series we did observe an increased frequency of TREM2 p.R47H in the PD patients from both populations. Interestingly, the frequency of TREM2 p.R47H was much lower in the Polish population and was not observed at all in the control subjects (Table 2). A combined analysis of all three PD patient-control series further confirmed the significant association of p.R47H with PD risk (OR = 2.67; p-value= 0.026).

**Table 2:** **Parkinson's disease association analysis of *TREM2 *variant c.140G>A (rs75932628; p.R47H)**

**Disorder (*n *)**		**Patient, *n *(%)**		**Control, *n *(%)**		**OR (95% CI)**		**p-value**^**a**^
Control	(1957)			8	(0.41)			
Combined	(1493)	16	(1.07)	8	(0.41)	2.670	(1.13, 6.33)	0.0256
North American PD	(683)	9	(1.32)	6	(0.45)	3.144	(1.1, 9.03)	0.0333
Irish PD	(367)	5	(1.36)	2	(0.54)	2.509	(0.47, 13.41)	0.2821
Polish PD	(443)	2	(0.45)	0		NA	NA	

^a^ Uncorrected p-values are shown.

There was no evidence of significant association in our North-American ALS series (OR = 1.47; p-value = 0.538), North-American PSP (OR = 1.54; p-value = 0.525) or the North-American ischemic stroke series (OR = 1.51; p-value = 0.569), although an increased frequency of p.R47H was observed across all diseases.

## Discussion

There are a number of pathways that are likely generalized across the myriad of neurodegenerative disorders, including the ubiquitin-proteasome system, mitochondrial dysfunction and the inflammatory response. TREM2 is an important player in the immune response and loss-of-function mutations in *TREM2* have been demonstrated to cause a spectrum of dementia-like phenotypes with or without bone cysts [3,5,7]. The recent identification of a rare TREM2 substitution (p.R47H) as a risk factor for AD now suggests that the protein plays an important role in neurodegeneration [5,8]. To further investigate TREM2 in disease we examined the frequency of p.R47H in a number of other disorders and identified a significant association with disease risk for FTD (OR 5.06) and PD (OR 2.67). These findings suggest that dysfunction of TREM2 may be involved in pathways that are underlying specific neurodegenerative processes, while the lack of association for PSP and ALS argues against a generalized immune dysfunction.

There is growing evidence to support the role of activated microglia pathways in neurodegeneration. A recent integrated systems approach by Zhang and colleagues examining genotype and whole-genome expression profiles of tissue from 1,647 late-onset AD and controls identified *DAP12* (*TYROBP*) as one of the key genes with differential expression in disease [9]. The authors also highlight their unpublished studies demonstrating that DAP12 is involved in amyloid-beta turnover and neuronal damage. TREM2 is a transmembrane signaling receptor expressed by microglial cells and functions with DAP12 to effect downstream signaling via β-catenin [10]. Recessive mutations in both TREM2 and DAP12 produce the clinical phenotype of Nasu-Hakola disease [3,11]. Interestingly, our group recently described mutations in the colony-stimulating factor receptor gene (CSF1R) in hereditary diffuse leukoencephalopathy with spheroids (HDLS) [12]. CSF1R is another member of this immune-related pathway and is a crucial mediator of microglial proliferation and differentiation in the brain. CSF1R also signals through β-catenin after stimulation by CSF1 [10]. HDLS can clinically manifest with personality and behavioral changes, dementia, parkinsonism, and related conditions, but not ALS, PSP or typical ischemic stroke. The reason for the selective presentation of HDLS patients with this set of clinical symptoms is currently unclear but may be dependent on specific downstream signaling pathways that are affected. If the p.R47H mutation in TREM2 affects the same pathway, this could provide a possible explanation for our lack of association with ALS, PSP and ischemic stroke.

This is the first report of an association of TREM2 p.R47H with neurodegenerative diseases other than AD and it is therefore important to consider potential confounding factors. While it is expected that in our clinical FTD series a subset of patients may in fact have a different pathological diagnosis, including AD [13,14], we observed a stronger odds ratio in our FTD series compared to previous reports in AD. Moreover, the association was strongest in our clinical series. The fact that multiple FTD families with recessive mutations in *TREM2* were also recently reported further strengthens a significant role for TREM2 in FTD. However, whether the concomitant amyloid pathology which is likely to exist in a substantial fraction of clinically diagnosed PD patients in our series could potentially contribute to the association of TREM2 p.R47H with PD is a question that we cannot address with our current study. Association studies in pathologically defined Lewy-body disease cohorts with and without amyloid pathology may be able to address this in future studies.

For years, research in these complex neurodegenerative diseases has focused on the study of common variants through genome-wide association studies; however, it is clear that common variants do not explain all the heritability described for these disorders. On the other hand, rare population variants with large or intermediate risk effect sizes have been more difficult to determine. As genetic technologies have now become more advanced the identification of intermediate risk variants such as p.R47H in TREM2 will also increase. The elucidation of these factors is extremely important and will provide further insights into the pathophysiology of disease and generate novel *in vitro* and *in vivo* systems in which to model the disease and screen therapeutics.

## Conclusions

Our study provides strong support for a role of the p.R47H variant in TREM2 in the etiology of FTD and PD. Together with the previous work implicating p.R47H in AD; these new findings suggest that the TREM2 p.R47H variant likely confers risk by altering the function of microglia, potentially by becoming less effective in the suppression of inflammation and clearance of apoptotic debris. Future work focused on the impact of p.R47H on TREM2 function is needed to clarify its precise role in these complex neurodegenerative disorders.

## Materials and methods

### Subjects

Demographics for the individual study groups are given in Table 3. All patients and controls were of Caucasian ancestry. Our ALS cohort (n = 765) consisted of 474 clinically diagnosed patients obtained from the Coriell Institute for Medical Research, 260 unrelated patients diagnosed with ALS according to El Escorial criteria from a consecutive clinical case series seen at the Mayo Clinic Jacksonville (MCJ) ALS Center, 3 patients from the University of California, San Francisco (UCSF), one patient from Northwestern University Feinberg School of Medicine.

**Table 3:** Demographic data of patients and controls included in the study

**Series**	***n***	**Age (years)**	**Females (%)**
Controls	1957	63.6 ± 15.7	54.6
North American	1324	64.0 ± 13.1	54.5
Irish	370	66.0 ± 22.2	63.2
Polish	263	59.5 ± 15.5	47.5
Amyotrophic lateral sclerosis	765	59.5 ± 12.1	42.5
Frontotemporal dementia	609	66.6 ± 10.3	47.5
Progressive supranuclear palsy	772	74.8 ± 7.7	43.3
Ischemic Stroke	448	66.0 ± 14.6	40.8
Parkinson's Disease	1493	59.7 ± 12.4	39.6
North American	683	63.8 ± 11.6	36.5
Irish	367	55.8 ± 12.1	44.7
Polish	443	56.4 ± 11.8	36.8

An additional 27 pathologically confirmed ALS patients were obtained from the MCJ brain bank. The FTD cohort (n = 609) included 477 clinically diagnosed FTD patients of unknown pathological subtype diagnosed with bvFTD, semantic dementia (SD) or progressive non-fluent aphasia (PNFA) and 132 patients with pathologically confirmed FTLD-TDP. The diagnoses of bvFTD, SD and PNFA were made using the Neary criteria [15]. FTD patients were ascertained from a total of 9 Centers between 1995 and 2010, including MCJ (n=179).

Mayo Clinic Rochester (n=134), Mayo Clinic Scottsdale (n=9), UCSF (n=117), Northwestern University Feinberg School of Medicine (n=21), Drexel University College of Medicine (n=24).

University of Texas Southwestern Medical Center (n=6), University of British Columbia, Canada (n=12), University of Western Ontario, Canada (n=42), and the MCJ brain bank (n=65). Importantly all ALS and FTD patients were previously analyzed for the *C9ORF72* repeat expansion, whereas FTD patients were also screened for mutations in the microtubule associated protein tau (*MAPT*) and progranulin (*GRN*) and all mutation carriers were excluded. PD patients (n = 683) were ascertained at MCJ and clinically diagnosed with PD according to published criteria [16]. The PD replication cohort consisted of 367 clinical cases and 370 controls from Ireland, and 443 clinical patients and 263 controls from Poland. All PD patients were screened for Leucine-Rich Repeat Kinase 2 (LRRK2) G2019S and all mutation carriers were excluded. The PSP series was made up of 772 subjects from the MCJ brain bank with pathologically confirmed PSP. Our Ischemic stroke cohort contains 448 patients from the Ischemic Stroke Genetics Study [17]. Finally, a cohort of unrelated US Caucasian control individuals free of neurodegenerative diseases (n = 1324), with no overlap to our earlier reported *TREM2* control population, was also available for genetic association studies. The ethics review board at Mayo Clinic approved the study.

### Genotyping

Genomic DNA was extracted from peripheral blood lymphocytes or brain tissue using standard procedures. The *TREM2* variant in exon 2 c.140G>A (rs75932628**;** p.R47H) was genotyped using a TaqMan Allelic Discrimination Assay on an ABI 7900HT Fast Real-Time PCR system (Applied Biosystems, Foster City, CA, USA). Positive controls were included to confirm assays were optimized for both alleles. Data analysis was performed using SDS 2.2.2 software. Positive or ambiguous results in the TaqMan assay were also confirmed and resolved via Sanger sequencing [18].

### Statistical analysis

The association between the *TREM2* variant rs75932628 (p.R47H) and each neurodegenerative disease was evaluated using a logistic regression model adjusted for age (age at final diagnosis for patients and age at blood draw for controls) and gender, where odds ratios (ORs) and 95% confidence intervals (CIs) were estimated. Given the low minor allele frequency and lack of homozygosity we examined association for TREM2 p.R47H and disease under a dominant model. P-values ≤ 0.05 were considered statistically significant and analyses were performed using PLINK (http://pngu.mgh.harvard.edu/purcell/plink/) [19].

## Competing interests

The authors declare that they have no competing interests.

## Authors’ contributions

SR carried out the molecular genetic studies, performed the statistical analysis and drafted the manuscript. BM and MB carried out the molecular genetic studies. TL, EF, WWS, KJH, CL-H, AK, EHB, CL, KAJ, DSK, CLW, RC, IRM, BLM, MB-J, GO, AK-W, MB, SGY, RCP, NE-T, RJU, JFM, KBB_,_ BFB, NRG-R, ZKW and DWD made substantial contributions to acquisition of patient material and data. RR and OAR conceived of the study, obtained study funding, participated in its design and coordination and drafted the manuscript. All authors read and approved the final manuscript.
